# Combined intravitreal dexamethasone and bevacizumab injection for the treatment of persistent diabetic macular edema (DexaBe study): a phase I clinical study

**DOI:** 10.1186/s40942-023-00449-w

**Published:** 2023-03-03

**Authors:** Francyne Veiga Reis, Pedro Dalgalarrondo, José Edisio da Silva Tavares Neto, Murilo Wendeborn Rodrigues, Ingrid U. Scott, Rodrigo Jorge

**Affiliations:** 1 Department of Ophthalmology, Ribeirão Preto Medical School, University of São Paulo, 3900, Bandeirantes av., Campus, 12fl., Ribeirão Preto, São Paulo 14048-900 Brazil; 2 Departments of Ophthalmology and Public Health Sciences, Penn State College of Medicine, Hershey, PA USA

**Keywords:** Diabetic retinopathy, Intravitreal injection, Aqueous solution of dexamethasone, Bevacizumab, Diabetic macular edema, Optical coherence tomography

## Abstract

**Purpose:**

The aim of this study is to investigate the safety of combined intravitreal injection of dexamethasone aqueous-solution (IVD) and bevacizumab (IVB) in patients with refractory diabetic macular edema (DME) and its effect on intraocular pressure (IOP), best-corrected visual acuity (BCVA) and central subfield thickness (CSFT).

**Methods:**

This prospective study included 10 patients (10 eyes) with DME refractory to laser photocoagulation and/or anti-vascular endothelial growth factor (anti-VEGF) therapy. A complete ophthalmological examination was performed at baseline, during the first week of treatment, and monthly through week 24. Therapy consisted of monthly injections of combined IVD and IVB “pro re nata” (PRN) if CST > 300 µm. We investigated the impact of the injections on intraocular pressure (IOP), cataract development, Early Treatment Diabetic Retinopathy Study (ETDRS) best corrected visual acuity (BCVA), and central sub-foveal thickness (CSFT) measured by spectral-domain optical coherence tomography (OCT).

**Results:**

Eight patients (80%) completed 24 weeks of follow-up. Compared to baseline, mean IOP increased significantly (p < 0.05) and anti-glaucomatous eye drops were necessary for 50% of the patients, CSFT was significantly reduced at all follow-up visits (p < 0.05), although mean BCVA showed no significant improvement. One patient developed dense cataract progression and another showed vitreoretinal traction at week 24. No inflammation or endophthalmitis was observed.

**Conclusion:**

Treatment of DME refractory to laser and/or anti-VEGF therapy with combined PRN IV dexamethasone aqueous solution and bevacizumab was associated with adverse effects related to the use of corticosteroids**.** However, there was a significant improvement in CSFT meantime best-correct visual acuity remained stable or improved in 50% of patients.

## Background

The effect of intravitreal dexamethasone aqueous solution in combination with intravitreal bevacizumab on intraocular pressure (IOP), cataract development, visual acuity, and macular thickness was investigated in patients with diabetic macular edema refractory to laser and/or anti-vascular endothelial growth factor therapy. A transient increase in IOP was observed in 5/10 patients and cataract progression in 2/10. There was a significant reduction in central subfield thickness (CSFT) measured by optical coherence tomography, and best-corrected visual acuity was stable or improved in 50% of patients.

## Introduction

Diabetic macular edema (DME) is the leading cause of reduced central vision in patients with diabetic retinopathy (DR) and has been reported in 26.1% of persons with more than 20 years of type II diabetes [1]. Persistent hyperglycemia is responsible for the breakdown of the blood-retinal barrier and the release of inflammatory and angiogenic factors such as cytokine activation, vascular endothelial growth factor (VEGF), tumor necrosis factors, interleukins, angiopoietins, protein C kinase, and final products of glycation [2]. Increased amounts of VEGF and inflammatory are intrinsically related to the pathophysiology of diabetic retinopathy and are responsible for the onset and perpetuation of macular edema as well as for the formation of compensatory neovessels to subsequent ischemia in the more advanced phases of DR. Due to the involvement of cytokines and VEGF in the inflammatory cascade of DME [3], periocular or intravitreal corticoids/corticosteroids such as dexamethasone and triamcinolone were widely used to treat DME before anti-VEGF agents became available [4].

Dexamethasone is a corticosteroid with a potent and rapid anti-inflammatory action, and triamcinolone is a synthetic corticosteroid with a prolonged anti-inflammatory action. Both agents have been shown to reduce DME and improve visual acuity (VA) on a short-term basis [4]. Despite their effectiveness and low cost, both agents are associated with the development/progression of cataracts and increased intraocular pressure (IOP), limiting the use of these drugs for ocular diseases [5]. However, low doses of corticosteroids have been shown to have similar therapeutic effects as higher doses with fewer complications, as more recently observed with a slow-release biodegradable implant of 0.7 mg dexamethasone (Ozurdex) [6].

Anti-VEGF therapy is currently the gold standard treatment for DME, and despite the structural differences between ranibizumab (Lucentis), aflibercept (Eylea), and bevacizumab (Avastin), their clinical effects in patients with DME have been reported to be, on average, similar [7]. In the literature, many reports and clinical trials have shown that anti-VEGF therapy alone is more effective in reducing the central macular thickness and improving visual acuity due to diabetic macular edema [7–10] as also in improving the level of retinopathy due to VEGF inhibition when compared with anti-VEGF therapy alone or with/without laser photocoagulation [7–10].

However, although reducing DME with anti-VEGF therapy is quite effective, many patients experience persistence or recurrence of macular edema even with repeated injections [7–10]. The high cost of these medications and the need for repeated injections is an important factor for the Unified Health System (SUS) in Brazil [11] having a high impact on public and private health systems also worldwide [12, 13].

Intravitreal corticosteroid therapy acts on the inflammatory cascade and has an important action in reducing the progression and severity of RD by reducing macular edema [14]. When associated with anti-VGEF, combined treatment of steroids and anti-VEGF can reduce the injection burden, [13, 14] and significantly elongate the treatment interval [15] and hence the cost of treatment.

Therefore, the aim of the current study was to evaluate the safety of the combination of the lowest-cost intravitreal anti-VEGF medication (bevacizumab) and an inexpensive steroid (dexamethasone solution) for the treatment of refractory DME, in addition to analyzing the anatomical and functional effects of this combined therapy in patients with DME.

## Materials and methods

### Study design

A prospective phase I clinical study was conducted according to the Declaration of Helsinki, after approval by the Research Ethics Committee of the University Hospital, Faculty of Medicine of Ribeirão Preto, University of São Paulo (HCFMRP-USP). All patients with refractory diabetic macular edema that presented between July/2019 and April/2020 at the Retina and Vitreous section of HCFMRP-USP were invited to participate in the study and gave written informed consent.

### Study population

Inclusion criteria were: (1) patients aged ≥ 18 years with type 1 or 2 diabetes mellitus; (2) presence of DME defined as a central subfield thickness (CSFT) > 300 µm, confirmed by spectral domain OCT (Spectralis: Heidelberg, Germany), for > 6 months with no response to previous treatments with anti-VEGF agents and/or laser and no previous ocular surgery other than cataract; (3) Early Treatment Diabetic Retinopathy Study (ETDRS) best-corrected visual acuity (BCVA) between 20/32 and 20/400. Exclusion criteria were: (1) aphakia or cataract surgery within the previous 4 months; (2) severe capillary loss determined by fluorescein angiography (FA); (3) changes in the vitreoretinal interface or vitreomacular traction detected by OCT; (4) proliferative DR with high risk characteristics; (5) any clinical condition that would prevent visualization of the fundus or patient follow-up; (6) glaucoma; (7) thromboembolic events (including acute myocardial infarction and cerebrovascular accidents) occurring less than 3 months before recruitment; (8) medical or psychological conditions that would prevent the patient from giving written informed consent; (9) significant and uncontrolled disease that, in the opinion of the investigator, might interfere with the study; (10) pregnancy or breastfeeding, and (11) participation in another clinical trial within the previous 30 days.

### Baseline and follow-up evaluations

After the determination of study eligibility, patients underwent comprehensive ophthalmological examination at the baseline visit, and at one week and monthly through week 24 post-injection. Study visits included logMAR BCVA measurements, anterior biomicroscopy for verification of anterior chamber and anterior vitreous cells and flare, applanation tonometry, dilated fundus examination by indirect ophthalmoscopy, fluorescein angiography and spectral domain OCT (Spectralis^®^, Heidelberg Engineering, Heidelberg, Germany) imaging. Glycosylated hemoglobin (HgbA1c) testing was performed at baseline.

A significant increase in IOP was defined as an increase of more than 5 mmHg compared to baseline. The decision to start IOP-lowering treatment was made if IOP was ≥ 20 mmHg, sustained for at least 2 consecutive visits. Lens opacity was classified as cortical, nuclear or subcapsular and graded from 1 to 4. The decision to perform cataract surgery was made after a discussion with the patient, taking into consideration the level of vision of both the affected and the contralateral eye.

OCT examinations were performed using the Spectralis^®^ HRA + OCT image system (Heidelberg Engineering, Germany). The center of the OCT scan was determined at baseline by the center of the fovea based on patient fixation. In subsequent visits, automatic follow-up from the Heidelberg machine was used to scan the same macular region from the previous visit. The strategy for analysis of CSFT was based on a grid thickness map automatically generated by the software.

### Treatment protocol

Patients were prepared with the application of topical 10% iodine-povidone to the eyelids and 5% iodine-povidone eye drops to the conjunctival site of injection. Intravitreal injection of bevacizumab (1.25 mg in 0.05 mL; Hoffmann-La Roche Ltd., Basel, Switzerland) and intravitreal injection of dexamethasone aqueous-solution (200 μg in 0.05 mL) were each administered with a BD Ultra-FineTM 29G½” disposable syringe needle, via the pars plana (3.5 mm from the limb) under topical anesthesia. One 250 mg acetazolamide tablet (Diamox^®^) was administered orally one hour before the procedure and the dose was repeated 4 h after both intravitreal injections. After the procedure, perfusion of the optic nerve was evaluated by indirect binocular ophthalmoscopy, with paracentesis of the anterior chamber considered in cases of poor perfusion. The patients were instructed to apply antibiotic eye drops (0.5% moxifloxacin), one drop every 4 h to the injected eye, starting 24 h before the injection for prophylaxis and continuing the application for 5 days after the injection.

Clinical ophthalmological evaluations including the same assessments as performed at baseline were performed at 1, 4, 8, 12, 16, 20, and 24 weeks after injections of bevacizumab and dexamethasone. Additional intravitreal injections of the combined drugs were administered if CSFT > 300 μm.

### Outcome measures

Safety outcomes such as mean intraocular pressure, signs of intraocular inflammation (anterior chamber cells or flare), progression of cataract and best-corrected visual acuity were verified prospectively. Although it does not represent a safety outcome, central subfield thickness was also checked prospectively.

### Statistical analysis

Data are reported as mean ± standard deviation (SD). Analysis of variance (ANOVA) followed by the Tukey test for multiple comparisons was performed. Paired means were compared by the Student t-test. Correlation analysis was performed using the Pearson correlation coefficient (r). The Origin Pro software version 8.6 was used for all analyses, with the level of significance set at p < 0.05.

## Results

A total of 10 eyes of 10 patients with chronic DME (> 6 months) unresponsive to previous treatments with anti-VEGF agents and/or laser were treated with intravitreal injections of bevacizumab and dexamethasone at baseline and PRN during the 24-week study period. Eight patients (8 eyes; 80%) completed all follow-up visits through week 24. Two patients (2 eyes; 20%) did not complete the 24th visit of the study for the following reasons: progression of cataract occurred in patient 1 and precluded tomographic quantitation of CSFT; patient 3 developed increased CSFT associated with vitreomacular traction. Both of these patients demonstrated improvement in BCVA and CSFT after phacoemulsification and pars plana vitrectomy, respectively.

The demographic characteristics of the 10 patients are presented in Table 1. Six (60%) patients were women; the mean age was 66.8 ± 3.5 years and all participants had type II diabetes, with a mean HgbA1c of 8.2 ± 1.5%.

**Table 1:** Patients demographics and baseline characteristics

Patient	Sex	Age (years)	DM duration (years)	HbA1c	Eye	DME duration (years)	BVCA(logMar) baseline	IOP baseline (mmHg)	CSFT (μm) baseline	Lens Opacity Score	Laser PRP/ST	Anti-VEGF	IVTA
1	M	69	8.0	9.2	OS	4	1.30	11	479	2 + /4 +	Yes	Beva	Yes
2	M	70	7.0	8.5	OS	3	1.10	15	587	1 + /4 +	Yes	Beva	Yes
3	M	70	10.0	8.0	OS	2	0.40	13	612	1 + /4 +	Yes	Beva	No
4	M	69	15.0	8.5	OD	2	0.40	17	514	1 + /4 +	Yes	Beva	No
5	F	70	16.0	9.1	OD	3	1.10	16	583	1 + /4 +	Yes	Beva	Yes
6	F	66	12.0	7.5	OS	3	0.70	16	586	1 + /4 +	Yes	Beva	Yes
7	F	60	9.0	9.5	OS	2	0.40	17	393	1 + /4 +	Yes	Beva	No
8	F	63	8.0	9.0	OD	2	0.40	16	387	1 + /4 +	Yes	Beva	No
9	F	64	12.0	9.0	OS	2	0.30	16	356	1 + /4 +	Yes	Beva	No
10	F	67	10.0	9.5	OD	2	0.30	12	306	1 + /4 +	Yes	Beva	Yes
***Mean***		***66.8***	***10.7***	***8.7***		***2, 3***	***0.64***	***14.9***	***480.3***	***1.1***			
***SD***		***3.5***	***2.9***	***0.6***			***0.38***	***2.1***	***112.4***				

*DM* diabetes mellitus, HbA1c

Those values correspond to: mean and mean standart deviation of age (years), DM duration in years, HbA1c, DME duration in years, BCVA logMar, IOP (mmHg), CFST in μm and the mean of lens opacity score

### Intraocular pressure

**Fig. 1:**
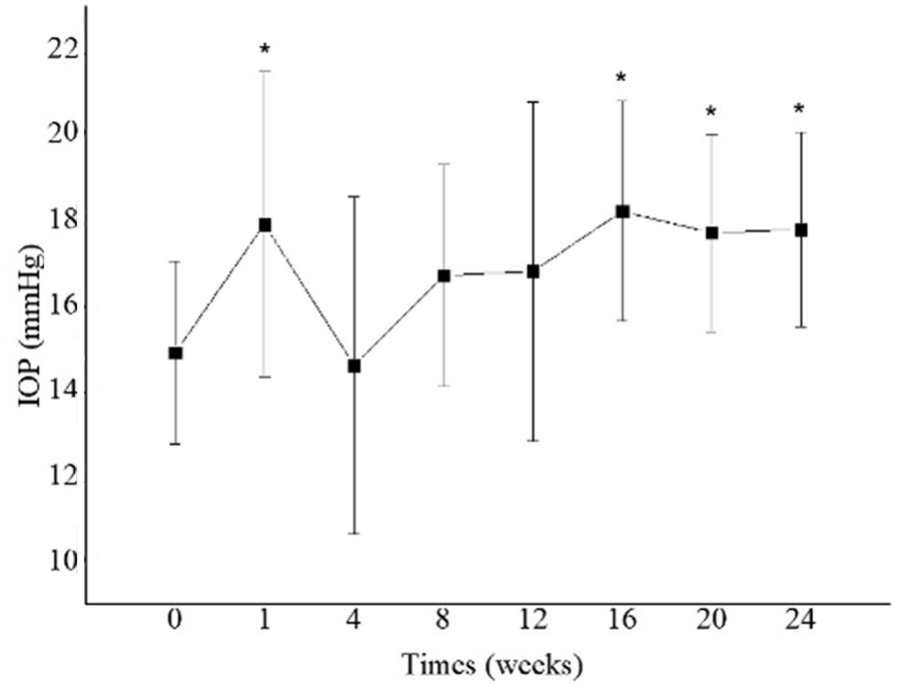
Mean intraocular pressure at baseline and during the 24 week-follow-up study period. *Indicates statistically significant change compared to baseline (p < 0.05). There was a significant increase on IOP at weeks 1, 16, 20 and 24 compared to baseline values

At baseline, mean ± SD IOP (mmHg) was 14.9 ± 2.1, and at Week 1: 17.9 mmHg ± 3.6 (p = 0.01); week 4: 14.6 mmHg ± 3.9 (p = 0.81); week 8: 16.7 mmHg ± 2.6 (p = 0.07); week 12: 16.8 mmHg ± 4.0 (p = 0.17). From week 16 through week 24, there was a significant increase in mean IOP: week 16: 18.2 mmHg ± 2.6 (p = 0.008); week 20: 17.7 mmHg ± 2.4 (p = 0.04); week 24: 17.8 mmHg ± 2.3 (p = 0.01). The highest mean IOP level occurred at week 1, with a mean ± SD IOP of 17.9 ± 3.6, which was significantly higher than at baseline (p = 0.01) (Fig. 1).

At week 1, three patients (3/10, 30%) had an IOP elevation > 5 mmHg compared to baseline. At week 12, one patient (no.7) presented with an IOP of 24 mmHg IOP, and an IOP-lowering medication (brimonidine) was initiated with prompt control of the condition. At week 16, five patients presented with IOP ≥ 20 mmHg that persisted to week 20 (50%), and IOP-lowering treatment was initiated; only one patient was taking more than one IOP-lowering medication (brimonidine + timolol) at the end of this study. None of the patients who developed IOP elevation needed surgery.

### Other adverse events

No patient had anterior chamber cells or any evidence of intraocular inflammation throughout the study period. After week 16 and 3 injections of dexamethasone plus bevacizumab, patient 1 showed progression of preexisting moderate cortical cataract (2 + /4 +) to complete opacification of the lens (4 + /4 + , 10%), which precluded image capture by OCT; this patient underwent phacoemulsification with intraocular lens implantation, with an improvement of visual acuity from 20/100 at baseline to 20/40. Patient 3 had an increase in CST at week 20 due to the development of vitreomacular traction. The patient underwent pars plana vitrectomy with an improvement of VA from 20/400 at baseline to 20/50. At week 24, patient 4 presented a rupture arterial retinal macroaneurysm, which was not verified at baseline, with a significant increase of CSFT. Patient 8 also demonstrated cortical cataract progression (from 1 + /4 + to 3 + /4 +) at week 16.

### Best-corrected visual acuity

**Fig. 2:**
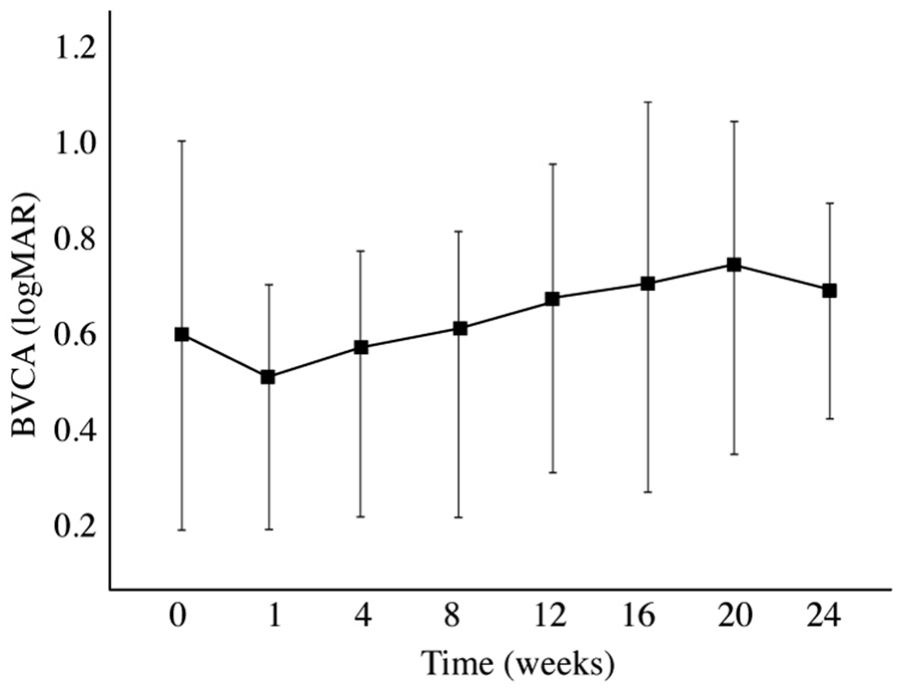
Mean logMAR best-corrected visual acuity during the 24-week follow-up period. There was only a trend towards significant improvement at week 1 (p = 0.08)

At baseline, the mean ± SD logMAR BCVA was 0.64 ± 0.38 [Snellen-equivalent = 20/87; ETDRS VALS (visual acuity letter score) = 53]. There was a trend towards BCVA improvement at week 1: mean ± SD logMAR BCVA was 0.49 ± 0.24 (Snellen-equivalent = 20/63; ETDRS VALS = 60) (p = 0.08). However, there was no significant improvement in mean BCVA at any follow-up visit (p > 0.05) compared to the baseline (Fig. 2). At week 24, five patients (50%) had stable or improved BCVA compared to baseline.

### Central subfield thickness

**Fig. 3:**
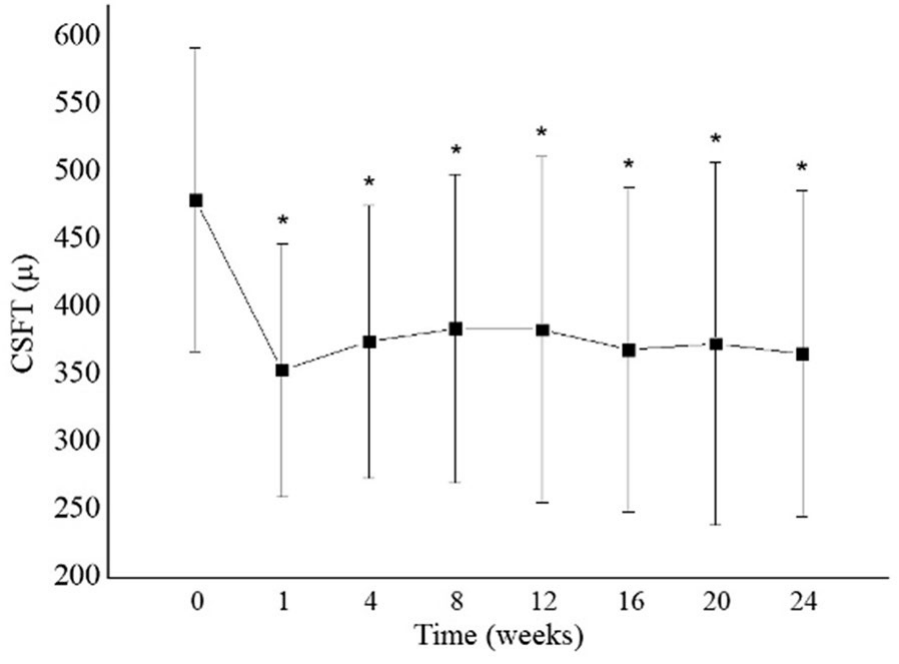
Mean central subfield thickness (CSFT) during the 24-week follow-up period. *Indicates statistically significant change compared to baseline (p < 0.05). There was significant improvement at all follow-up visits when compared to baseline

Mean ± SD CST (µm) was 480 ± 112 at baseline and significantly reduced at each of the follow-up visits to 355 ± 94 (week 1), 376 ± 101 (week 4), 385 ± 114 (week 8), 384 ± 128 (week 12), 370 ± 120 (week 16), 374 ± 135 (week 20), and 367 ± 121 (week 24). The maximum reduction from baseline in mean CST occurred at week 1 (Fig. 3).

### Number of injections

**Fig. 4:**
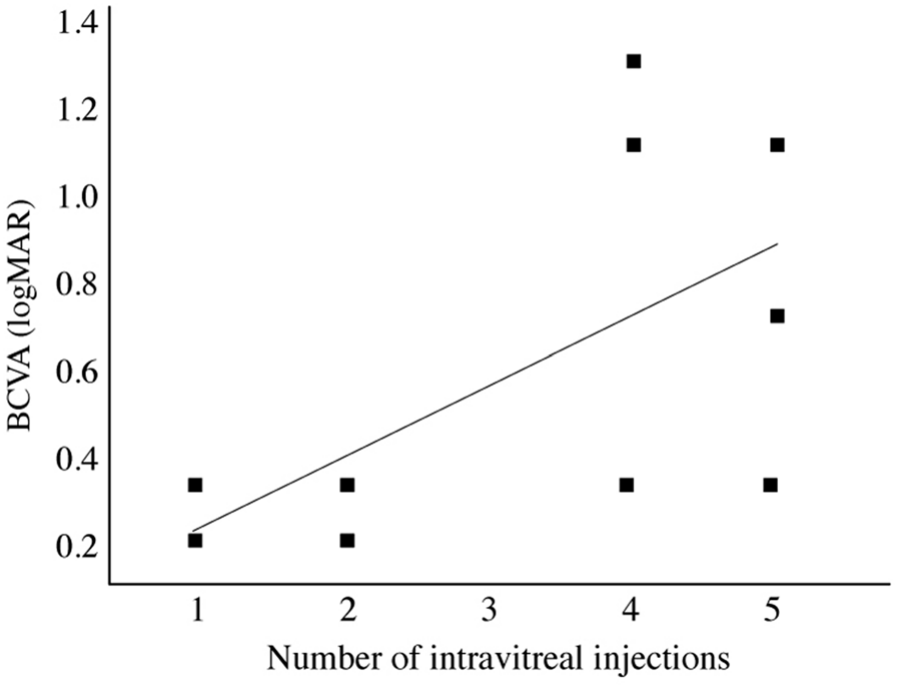
Mean baseline best-corrected visual acuity (BCVA) by the number of injections during the follow-up period. There is a moderate positive correlation between LogMAR baseline BCVA and the number of injections administered (r = 0.56). In other words, patients with worse baseline visual acuity required more intravitreal injections during the 24-week follow-up period

The mean number of intravitreal injections (combined treatment) was 3.3 out of a possible maximum of 5 during the 24-week study period. There was a moderate positive correlation between baseline BCVA and number of injections administered (r = 0.56), with a mean of 2.5 injections administered among patients with a baseline BCVA better than 20/50 (0.4 logMAR) compared to a mean of 4.5 injections among patients with a baseline BCVA worse than 20/100 (0.7 logMAR) (p < 0.05) (Fig. 4).

## Discussion

The primary objective of this phase I study was to investigate the safety of combined treatment with intravitreal dexamethasone and bevacizumab in a small sample of DME patients. For this reason, only 10 patients were enrolled and the focus was on safety parameters, such as IOP elevation, cataract development/progression, intraocular inflammation of anterior chamber and/or vitreous cells, and flare and BCVA changes. We did not include visual field or electroretinography (ERG) since intravitreal injections of dexamethasone and bevacizumab have been used separately for years for the treatment of several eye conditions [5, 6, 13, 14]. Bevacizumab has been used worldwide for neovascular age-related macular degeneration (AMD), DME, retinal vein occlusion, and intravitreal dexamethasone has been used for DME, retinal vein occlusion, uveitis, and endophthalmitis. We also assessed the efficacy parameters of CSFT and the number of intravitreal injections administered.

The combination of anti-VEGF and corticosteroid may lead to better results as shown in the RISE and RIDE trial and other clinical studies, than therapy with anti-VEGF alone since about 50% of patients with DME did not improve VA after 2 years of monthly treatment with intravitreal ranibizumab (IVR) [9, 10]. Similarly, in Protocol I conducted by the Diabetic Retinopathy Clinical Research Network (DRCR.net), nearly 40% of patients treated with IVR with focal macular grid laser, combined early or late, maintained DME after 2 years of treatment [10, 13]. Also, some evidence suggests that the late phase of DME may be due to more inflammatory activities than angiogenic factors [10, 16, 24]. These examples suggest that other factors that are independent and unresponsive to VEGF may be involved in cases of persistent DME [20], which reinforces the need for other alternative or combination therapies.

The known anti-inflammatory and VEGF-inhibiting properties of corticosteroids may potentiate the reduction of macular thickness in chronic cases of DME refractory to treatment with anti-VEGF medication alone [16, 17]. Prior clinical studies have investigated the use of intravitreal or subtenon administration of triamcinolone acetonide (TAAC) [17, 18], intraocular implant of dexamethasone (Ozurdex) [6, 19], or fluocinolone acetonide [20] and intravitreal injection of dexamethasone [21] in patients with DME.

Corticosteroids such as dexamethasone and triamcinolone acetate are known to downregulate cytokines and prostaglandins, inhibit leukostasis, leukocyte adhesion and transmigration, and inhibit the expression of growth factors such as VEGF [23]. Furthermore, they decrease vascular permeability, and subsequent leakage in the retina acting on the endothelium of the basal membrane of retinal capillaries and tight junctions [22, 24]. However, triamcinolone is more lipophilic and those crystals deposited more in the trabeculae, resulting in expressive increases in IOP and cataract formation [17–19, 25]. Because dexamethasone is less lipophilic, the sustained-release corticosteroid delivery system—Ozurdex^®^, 700 µg intravitreal dexamethasone implant (Allergan, Inc., Irvine, CA, USA) has been widely used as an alternative to DME treatment reducing the need for frequent intraocular injections and has shown significant long-term improvement in VA and DME persistent or resistant to anti-VEGF therapy, including in vitrectomized patients, who have a lower response to intravitreal therapies [19], with a mean time of action of approximately 4 months in real life. Despite having an effect on IOP and cataract formation, there is better IOP control with hypotensive eye drops and cataracts are usually observed after 3 consecutive implants. Despite all its effectiveness, Ozurdex^®^ is as expensive as anti-VEGF therapy for Brazil's public health system.

So, for the current investigation, dexamethasone aqueous solution was selected because dexamethasone has been demonstrated to have a sevenfold higher potency compared to TAAC because it the less lipophilic, a property that has been associated with a lower accumulation in the trabecular network and a lower impregnation of the crystalline lens, suggesting lower risks of IOP elevation and cataract formation/progression [21–23]. Further, dexamethasone aqueous solution acts on the inflammatory cascade and, associated with bevacizumab, reduces inflammatory factors meanwhile anti-VEGF promotes long-lasting sustained inhibition of VEGF. Moreover, the aqueous solution of dexamethasone was selected due to its much lower cost compared to the intravitreal implant of the same drug.

Despite intravitreal injections of dexamethasone phosphate were previously considered ineffective for DME probably because of rapid clearance from the vitreous and the short half-life of the drug [22, 25], Fonseca’s [26] results showed a significant reduction in macular thickness. Their short-term follow-up study aimed to investigate the effect and safety of 4 mg/mL dexamethasone solution during 28 days follow-up, and they find a significant reduction in macular thickness 3 days after 4 mg/mL dexamethasone solution injection and a significant reduction of macular thickness was observed between D0 and D3 (p = 0.008) and D0 and D7 (p = 0.021), while no significant reduction of was seen between D0 and D28 (p = 0.859). The study showed also a significant decrease in macular thickness (12.85% over baseline values for the entire cohort) 7 days after the intravitreal injection, whereas it was speculated that the action of dexamethasone would have an effect of only a few hours, demonstrating its short-time effect in reducing macular thickness [26]. However, these authors did not investigate the combination of intravitreal dexamethasone and anti-VEGF therapy. Therefore, we hypothesized that the intravitreal aqueous solution of dexamethasone would provide an acute inhibition of the inflammatory cascade and subsequent DME improvement, which could then be sustained by the longer-lasting effect of the anti-VEGF agent bevacizumab.

The association of corticosteroids and an antiangiogenic agent for DME treatment has been reported by some investigators using different drugs and formulations [1, 14, 18, 20, 27–29]. Faghihi et al. reported [28] results on macular thickness reduction and visual acuity improvement comparing single IVB therapy, combined IVB plus IVT or macular laser photocoagulation. After 16 weeks, the combined IVB plus IVT therapy revealed sustained and superior results of visual acuity improvement 0.1 logMAR (p < 0.001) compared to single IVB-therapy and laser therapy. Folgosa et al. [29] reported the effects of the use of IVT combined with IVB during a 24-week study for DME refractory to macular laser therapy where visual acuity improvement was near ± 0,2 LogMAR between the 4th and 8th week visit (p < 0.05) and macular thickness reduction was observed at 1, 4 and 8 weeks after treatment (CSFT reduction of 17%, 32% and 22%, respectively), with the results being statistically significant (p < 0.05) compared to baseline.

In our investigation, there was a significant mean IOP increase at weeks 1, 16, 20, and 24 compared to baseline, and 5 patients (5/10; 50%) developed IOP > 5 mmHg compared to the baseline visit and were treated with IOP-lowering eye drops. Elevated IOP and even “intractable” IOP are adverse events related to intravitreal or periocular steroids [30, 31]. Fonseca et al. did not observe significant IOP elevation in patients treated with a single dose of 40 μg, 120 μg, or 200 μg of intravitreal dexamethasone solution [26]. In their study with a 28-day follow-up, a maximum IOP rise of ± 1.89 mmHg occurred one day after intravitreal injection of 200 g dexamethasone aqueous solution. In our study, a maximal mean IOP increase of 3.0 mmHg occurred one week after injection of 1.25 mg bevacizumab in combination with 200 μg dexamethasone aqueous solution; the combination of anti-VEGF and steroid in our study likely contributed to a higher IOP peak in the short term, probably due to a longer follow-up (24 weeks) and repeated injections. In the IVB + IVT group of Faghihi’s study, only two eyes (2/41, 4.8%) developed elevated IOP, which was controlled with topical medication [28]. In an expanded two year follow-up of a randomized trial evaluating intravitreal 0.5 mg ranibizumab or 4 mg triamcinolone combined with focal/grid laser compared with focal/grid laser alone for diabetic macular edema, intraocular pressure and cataract surgery were more frequent in the triamcinolone + prompt laser group in a study by Elman et al. [16] In the study by Folgosa et al., there was a small but statistically significant IOP increase of 2.1 ± 0.6 mmHg, recorded only at the 4th week after treatment, but none of the patients needed IOP-lowering eye drops [29].

Cataract progression was observed in two patients (2/10, 20%) from our study in comparison to one (1/41, 2.4%) in the study by Faghihi et al. [28]. This difference may be due to the fact that, in our study, patients received a mean of three injections of combined anti-VEGF and dexamethasone while in the study by Faghihi et al. patients received only a single intravitreal injection of bevacizumab and triamcinolone (2 mg) [28]. In addition, our study had a longer follow-up (24 weeks versus Faghihi’s 16-week study). Fonseca’s [26] study was too short (1 month) for cataract detection and Folgosa [26] did not report cataract occurrence.

In our study, one patient (10%) developed vitreomacular traction 20 weeks after baseline. This patient did not have proliferative diabetic retinopathy, and there was traction only in the foveal region, probably due to vitreous syneresis and subsequent detachment of the posterior vitreous, which remained firmly attached to the foveal region. Bakri and Omar reported worsening of vitreomacular traction after a dexamethasone intravitreal implant in a patient with macular edema secondary to central retinal vein occlusion [32]. According to their theory, vitreomacular traction became more evident after fluid reabsorption and macular edema regression [32]. In our study, the patient who developed vitreomacular traction underwent pars plana vitrectomy, which improved logMAR BCVA from 0.1 to 0.4. Another patient in our study presented at week 20 with increased CST and decreased BCVA associated with exudation from a retinal arterial macroaneurysm that was not present at baseline evaluation. This patient had a long-standing history of systemic hypertension, which is a well-known risk factor for retinal arterial macroaneurysm. Despite these events, no other complications such as anterior chamber reaction or endophthalmitis were noted in this study regardless of the previously reported association of dexamethasone phosphate and a higher rate of endophthalmitis than with anti-VEGF agents (0.13% X 0.019%, respectively) [32].

The baseline BCVA of our study population was 0.64 ± 0.38 and was similar to the mean baseline BCVA of the combined injection groups in the studies by Faghihi et al. [28], Folgosa et al. [29], and Fonseca [26]: 0.77 ± 0.33, 0.72 ± 0.38 and 0.5 ± 1.4, respectively. Similar to the 200 μg group from Fonseca’s study, our patients did not show a significant increase in mean BCVA at any study visit [23]. On the other hand, Faghihi et al. [28] reported a 0.10 logMAR improvement in mean BCVA over 16 weeks in their study population. Folgosa et al. [29] reported a mean BCVA improvement of approximately 0.20 logMAR at week 8, but VA improvement was no longer observed at weeks 12 and 24. BCVA improvement differences may be related to the chronicity of macular changes and other factors such as lens status that was not well controlled in these small sample studies.

The mean baseline CSFT of our study population (480 ± 112 μm) was comparable to Folgosa’s study population (413 μm) and both are higher than that reported by Faghihi et al. for the IVB + IVT group (387 ± 154 μm) [28, 29]. At week 16, we observed a significant reduction in mean CST of 131 µm, which is more than the reduction observed by Faghihi et al. (102 ± 74 μm) at week 16 [28]. This may be because our study population had a higher baseline CSFT than observed in the former study, or may be related to the fact that we performed monthly PRN combined injections of dexamethasone and bevacizumab, while Faghihi et al. performed just one combined injection [28]. Similarly to Faghihi et al. Folgosa et al. observed a smaller CSFT reduction (78 μm) at 8 weeks, which was no longer present at 12 and 24 weeks, perhaps because only a single injection was administered to patients in that study [28, 29].

Regarding the number of injections, we observed that patients with better baseline VA required fewer injections than patients with worse baseline VA, despite the chronicity of macular edema. Dugel et al., when using anti-VEGF for persistent DME patients, associated or not with laser therapy also reported that patients with better baseline VA needed fewer injections in the first year [33].

A major limitation of our study is its small sample size. For this reason, findings of efficacy should be interpreted with caution. In the short term, the combination of PRN intravitreal dexamethasone and intravitreal anti-VEGF for DME treatment may significantly increase IOP and IOP-lowering drops may be required. Cataract is another potential adverse effect of this combined therapy, which seems otherwise safe to reduce macular edema. Further investigation with a larger number of patients is warranted.

## Data Availability

All data generated or analyzed during this study are included in this published article [and its Additional files].

## Abbreviations

VEGF: Vascular endothelial growth factor
DME: Diabetic macular edema
CSFT: Central sub-foveal thickness
IVD: Intravitreal injection of dexamethasone aqueous-solution
IVB: Intravitreal injection of bevacizumab
IOP: Intraocular pressure
BVCA: Best-corrected visual acuity
CSFT: Central subfield thickness
PRN: “Pro re nata”
IOP: Intraocular pressure
ETDRS: Early Treatment Diabetic Retinopathy Study
OCT: Optical coherence tomography
DR: Diabetic retinopathy
VA: Visual acuity
HCFMRP-USP: Faculty of Medicine of Ribeirão Preto, University of São Paulo
FA: Fluorescein angiography
logMAR: Logarithm of the minimum angle of resolution
HgbA1c: Glycosylated hemoglobin
SD: Standard deviation
ANOVA: Analysis of variance
VALS: Visual acuity letter score
ERG: Electroretinography
AMD: Age-related macular degeneration
IVR: Intravitreal ranibizumab
DRCR.net: Diabetic retinopathy clinical research network
TAAC: Triamcinolone acetonide
FVRC: Author Francyne Veiga Reis, M.D.
PD: Author Pedro Dalgalarrondo, M.D.
JESTN: Author José Edisio da Silva Tavares Neto, M.D.
MWR: Author Murilo Wendeborn Rodrigues, M.D., Ph.D.
IUS: Author Ingrid U Scott, M.D., M.P.H.
RJ: Author Rodrigo Jorge, M.D., Ph.D
